# Rapid monitoring of cropland primary productivity and shipping activity in Ukraine

**DOI:** 10.1371/journal.pone.0286637

**Published:** 2023-06-28

**Authors:** Michael Wellington, Petra Kuhnert, Roger Lawes

**Affiliations:** 1 CSIRO Agriculture & Food, Black Mountain, Australian Capital Territory, Australia; 2 Fenner School of Environment and Society, Australian National University, Acton, Australian Capital Territory, Australia; 3 CSIRO Data61, Dutton Park, Queensland, Australia; 4 CSIRO Agriculture & Food, Floreat, Western Australia, Australia; University of Strathclyde, UNITED KINGDOM

## Abstract

Ukraine is an important global exporter of grain, especially to several countries with vulnerable food systems. The war in Ukraine may disrupt global food supply by limiting the planting, growth, and harvest of crops, or disrupting grain supply logistics. We apply a novel statistical modelling approach to satellite images of cropland in Ukraine for fast inference and exploration of cropping patterns and their influences in challenging environments. We also present satellite-derived cargo shipping activity as an accompaniment to these outputs to better explore the outcomes. Cropland Gross Primary Productivity in 2022 was 0.25gC/m^2^ lower than the 2010–2021 baseline period (p < 0.001). Similarly, cumulative annual cargo shipping activity ports in the Odesa and Mariupol regions were 45% and 62% lower in 2022 than in 2021, respectively. This suggests that cropland primary productivity has suffered during the conflict, and reliance on a few key port areas introduces vulnerability to the value chain.

## Introduction

Ukraine is a major producer and exporter of wheat, barley, maize, and sunflower products [1]. Exports from Ukraine account for approximately 12% of traded grains globally [2]. Imports from Ukraine are heavily relied upon by African and south Asian countries with compromised food systems [3]. These countries are often assisted by the World Food Programme, which itself sources up to 40% of its grain from Ukraine [3–5]. Disruption to grain production and exports from Ukraine due to conflict with Russia can therefore have serious detrimental effects on the global food system.

The impacts of conflict in Ukraine on agricultural production and exports have been widely reported in the media. Detrimental impacts on the planting, growing, and harvesting of crops in Ukraine have been attributed to explosives in fields, conscription of farmers, inaccessibility of fertiliser, cropland fires from incendiaries, and other disturbances [6–8]. Further, the closure of Ukraine’s Black Sea ports and subsequent deal to reopen them has been widely reported [9].

The use of satellite monitoring technology has been suggested as a critical means of assessing production through conflicts [3]. Long-term trends in vegetation greenness and productivity can be related to crop production and have been used to estimate changes in crop yields in Ukraine during the war [10–12]. However, the very large data sets associated with satellite imagery make accommodating both spatial and temporal variation difficult. Numerous software packages exist that simplify trend estimation by computing trends on mean annual images [13], time series of pixels, or on spatial aggregations. Some approaches allow trends to be decomposed from repeating seasonal patterns [14, 15], though these also operate on time series of pixels or spatial aggregations. Importantly, these approaches seldom account for temporal and spatial autocorrelation, as identified by Ives et al. [13]. Further, machine learning methods and presentation of raw data summaries in previous satellite-based assessments of crop condition in Ukraine [12, 16, 17] do not quantify uncertainty in estimates of changes or trends.

We present a novel modelling framework which decomposes satellite image time series into seasonal patterns and inter-annual trends, accommodates spatiotemporal interactions, and their dependencies. The framework draws upon recent advances in Generalised Additive Models (GAMs) that allows for much larger representations of additive and latent Gaussian processes applied to very large datasets [18, 19]. In applying this approach to Ukraine at the national scale, we demonstrate its capacity to capture non-linear inter-annual, seasonal, and spatial dynamics in a statistically robust manner. Further, the model can be adapted to test hypotheses about temporal and spatial changes, such as altered cropland primary productivity post-invasion.

## Methods

### Extraction of cropland Gross Primary Productivity

Gross Primary Productivity (GPP), as an estimate of the photosynthetic rate or biomass production of vegetation, is a widely applied proxy for crop production and underpins numerous remote sensing yield assessments [20, 21]. The estimation of GPP combines satellite-derived reflectance information, such as vegetation greenness, with climatic conditions [20, 22]. GPP therefore accommodates more environmental limitations to crop growth than observational measures, such as vegetation indices [20]. However, there is uncertainty associated with any remote sensing product including modelled GPP. Additionally, cropland GPP may not always linearly relate to variation in harvestable product, such as grain, due to variation in harvest index (harvested biomass as a proportion of total crop biomass) [23]. Furthermore, uncertainty in planted and harvested area means that GPP based assessments may be somewhat confounded by unharvested crops or weed growth in fallow or abandoned fields. GPP is therefore a useful but imperfect proxy for crop production.

GPP data were downloaded from the Moderate Resolution Imaging Spectroradiometer (MODIS) 8-day (MOD17A2H) product from Google Earth Engine with an R interface. The `Gpp’ layer from the MODIS/006/MOD17A2H image collection [22, 24, 25] was selected with the ‘select’ function in the ‘rgee’ package [25]. GPP data extracted from the 1^st^ of January 2010 through to the 31^st^ of December 2022 were clipped to the bounds of Ukraine using the Food and Agriculture Organisation Global Administrative Unit Layers Country Boundaries product [24], then clipped to croplands using the Global Food Security Analysis Dataset [26]. Extent clipping was performed with the ‘clip’ command from the R Google Earth Engine (rgee) package [25].

The GPP image collection for cropland in Ukraine for the selected period was then converted to an R dataframe for the purpose of exploring spatio-temporal trends in GPP and how these trends altered (if any) during conflict between Russia and Ukraine. This was executed with the ‘to_dataframe’ command in the ‘rgee’ package [25]. Attributes from the resulting dataframe were used to create longitude, latitude, and date columns. The date column was split into additional year, month, and day columns using the ‘lubridate’ R package [27]. Finally, a pre and post invasion term, ‘war’, was added which labelled each observation as either pre or post the 24^th^ February 2022. This gave a final dataframe of 1,292,171 observations and 8 columns: longitude, latitude, date, GPP, year, month, day, and ‘war’.

#### Spatio-temporal modelling framework

We explored a generalized additive model (GAM) for capturing the spatio-temporal dynamics of GPP, with a view of understanding trends in cropland GPP in Ukraine. GAMs have a long history in being applied to practical problems in domains such as epidemiology, agriculture, and the environment [28–30]. They represent a flexible, non-parametric statistical method that captures non-linear relationships and complex patterns using smooth functions driven by the data. GAMs provide a statistically interpretable output for inference, contrasting machine learning ‘black box’ methods used in Lin et al. [12]. Further, the flexibility of the GAM allows it to model a wide range of complex relationships in the data, including spatial and temporal relationships and their interactions [31]. More specifically, a GAM can be considered a special case of a Gaussian process because it uses smooth functions such as splines to accommodate a wide range of complex, non-linear relationships in the data, making it a powerful tool for modelling complex phenomena. More recently, Wood et al. [19] have extended the method and developed a scalable additive model that is capable of efficiently and quickly fitting smoothers to gigabytes of data. This was achieved by ‘discretizing’ covariates for much faster model computation [18]. This recent advancement has meant that GAMs are now strong competitors [13–15, 32] for spatio-temporal modelling and inference at scale, with results in Wellington et al. [33] showing that it is possible to run a spatio-temporal GAM on millions of observations in a matter of minutes. While machine learning approaches may provide superior computational efficiency, the fast implementation of GAMs makes it a very efficient method for conducting statistical inference on spatio-temporal data with flexible smooths.

The modelling framework we propose is outlined in Wellington et al. [33] and appears below. The method uses penalized regression splines to capture spatial, temporal and seasonal relationships in remotely sensed GPP. We use an implementation of the mgcv R package [34] that provides the computational speed necessary to fit the model [19]. More specifically, the model can be written as:

$$\begin{array}{c} z_{i}\sim N\left(\mu_{i},\varphi \right)\\ \mu_{i}=\beta +f_{1}\left(y_{i}\right)+f_{2}\left(m_{i}\right)+f_{3}\left(s_{i}\right)+f_{4}\left(y_{i},m_{i}\right)+f_{5}\left(s_{i}y_{i}\right) \end{array}$$ (1)

where *z*_*i*_ represents a measure of GPP at the *i*-th pixel, assuming a Gaussian distribution with mean, *μ*_*i*_ and scale parameter, *φ* and *β* is the intercept term, and *f*_*j*_(*j* = 1, … 5) represents smooth functions to be estimated that capture non-linear temporal and spatial relationships through combinations of *m*_*i*_, *y*_*i*_, and *s*_*i*_, that represent month, year and spatial terms (latitude and longitude), respectively. The mean represents a function of smooth terms (*f*_1_ − *f*_5_) that capture linear and non-linear effects, temporal, and spatial relationships along with seasonal components. A cubic regression spline was used for *y* with the basis dimension set to the number of unique years [39]. A cyclic cubic regression spline was used for *m*, with the basis dimension set to 12 [40]. The spatial smooth term, *s*, was represented by a Gaussian process smooth and basis dimension of 50 [41] to accommodate spatial dependencies inherent in the remote sensed data. Each interaction term was fit with the same specification.

The model was fit using the ‘mgcv’ package in R version 4.1.3 [35] and extended to include an autoregressive (AR) error term of order 1 to examine the temporal dependence using the methods outlined in Section 2.1, in Wood et al. [36], where the covariance matrix of.*φ* is *ε*Σ, where Σ represents an auto-regressive AR(*ρ*) correlation matrix. The correlation term, *ρ*, was estimated using a one-dimensional search that required the model to be refitted at each computational stage. This fast alternative to the estimation of *ρ* which is then provided as a “plug-in” estimate to the model is outlined in Van Rij et al. [37]. While other error structures could be explored, we found that fitting an AR1 term in this model sufficient for accommodating the temporal dependence.

Temporal and spatial dependence was investigated using autocorrelation plots and variograms, which can be viewed in the online analysis vignette (https://mickwelli.github.io/Ukraine-crops/articles/Ukraine-GPP-analysis.html). An R package for reproducing the analysis presented in this manuscript can be accessed through the authors github account (https://github.com/mickwelli/Ukraine-crops) [38] along with an accompanying vignette that outlines the analysis and supporting diagnostics to assess the fit of the model (https://mickwelli.github.io/Ukraine-crops/articles/Ukraine-GPP-analysis.html) [38]. The residual spatial semivariance plot and temporal autocorrelation plot show that the model effectively captures all spatial and temporal variance in the GPP data.

We also compared GPP values post-invasion on the 24^th^ February 2022 to pre-invasion by including this in our statistical inference framework as a factor. This was achieved by modifying Eq 1 to:

$$\begin{array}{c} z_{i}\sim N\left(\mu_{i},\varphi \right)\\ \mu_{i}=\beta +w_{i}+f_{1}\left(m_{i}\right)w_{i}+f_{2}\left(s_{i}\right)w_{i}+f_{3}\left(s_{i}m_{i}\right)w_{i} \end{array}$$ (2)

where *z*_*i*_ is GPP at the *i*-th pixel assuming a Normal distribution with mean *μ*_*i*_ and scale parameter φ, β is an intercept, *w*_*i*_ is a factor representing either pre or post the Russian invasion of Ukraine, and *m*_*i*_ and *s*_*i*_ represent month and spatial (longitude and latitude) terms, respectively. This model was fitted with the same smooth parameters and autoregressive terms listed for Eq 1, above. In addition, after accounting for spatial and temporal variation in the data, the effect of the ‘war’ term, *w*_*i*_, was directly estimated to highlight direction of GPP change and its associated significance.

#### Validation with real crop production data

GPP is a measure of vegetative biomass production. Satellite based estimates of GPP underpin numerous large-scale crop production assessments [20, 21]. However, there is some uncertainty associated with modelled GPP and it may not always relate well to crop production [23], so tests against real production data should confirm agreement where possible.

Total crop production data in tonnage from 2010 to the latest available year, 2020, were downloaded from the FAOSTAT database [39] as a.csv file for comparison with the GPP trend analysis. Ukraine was selected from the country list and barley, maize, wheat, rapeseed, potatoes, and sunflower seed were selected from the crop production list. The crop types selected were based on those that appeared in the UN Food and Agriculture Organisation Global Information and Early Warning System on Food and Agriculture factsheet crop calendar for Ukraine [1]. The downloaded file was loaded into the R programming environment and plotted against the summed effect of year on GPP using the ‘ggplot2’ package [40]. Predictions from the GAM for the main effect of year were extracted from the model using the ‘predict.gam’ function that appears in the ‘mgcv’ R package [34]. A Shapiro-Wilk test for normality was performed on both the annual GPP prediction terms and total tonnes of crop produced using the ‘shapiro.test’ function from the R ‘stats’ package to confirm normality prior to a correlation test. A one-sided Pearson correlation test was conducted using the ‘cor.test’ function in R to determine the strength of the relationships between the predictions from the model and total crop production data. This testing process is shown in online R vignettes (https://mickwelli.github.io/Ukraine-crops/articles/Ukraine-GPP-analysis.html) [38].

#### Exploration of smooth terms from the GAM

The smoothed additive terms from the model were explored through plots produced using the `plot_smooth’ function from the ‘itsadug’ package [37], an R package to allow exploration of GAM outputs. This approach was used to explore the yearly and seasonal terms in the model and how they compared pre- and post- invasion periods. Plotting was achieved using the ‘ggplot2’ R package.

Additionally, an indicative crop calendar was sourced from the UN Food and Agriculture Organisation Global Information and Early Warning System on Food and Agriculture factsheet for Ukraine [1]. This was adapted to a ‘ggplot2’ plot and plotted adjacent to the summed effect of month on GPP for inference of GPP patterns alongside the crop calendar.

Spatial patterns in cropland GPP pre and post invasion were plotted using the ‘fvisgam’ function in the ‘itsadug’ package [37]. The GPP estimates and standard errors produced by this function were passed to ‘Vizumap’, which allows presentation of uncertainty on maps [20]. A bivariate colour palette was produced using the ‘Vizumap’ ‘build_palette’ function with terciles for both GPP estimates and standard errors, and colour hex codes were added to the dataframe using the ‘build_bmap’ command. The ‘geom_raster’ function in ‘ggplot2’ was used to plot the spatial estimates and uncertainty with the ‘fill’ argument set to the hex code column. All data and code used to produce these plots are available in online R vignettes (https://mickwelli.github.io/Ukraine-crops/articles/Ukraine-GPP-analysis.html) [38].

#### Analysis of cargo shipping

Cargo shipping data for each month of 2021 and January to August 2022 were downloaded from the European Marine Observation and Data Network (EMODNet) [41] in 1-km resolution. Data from the downloaded.tif files were loaded into R and stacked together using the ‘stack’ command in the ‘raster’ [42] package. The raster stack object was reprojected to a WGS84 projection using the ‘projectExtent’ command and cropped to longitude ranging from 25.5 to 43.5 and latitude ranging from 40.1 to 48.1 with the ‘crop’ function of the ‘raster’ package.

Cumulative route densities for each port area were extracted from the monthly raster stack object by extracting mean values from a 20km radius around coordinates representing each port zone. The coordinates used for each location are presented in Table 1. The mean values were extracted using the ‘extract’ function from the ‘raster’ package with the ‘buffer’ argument sent to 20,000m. Results were stored in a dataframe with columns for port, date, year, month, and day which were extracted using the ‘lubridate’ R package [27, 42]. The ‘cumsum’ function from the ‘tidyverse’ package was used to calculate the cumulative sum of shipping activity in each year, and the result was plotted using ‘ggplot2’. The percentage change in cumulative route density was calculated by dividing the difference between cumulative values in December 2022 and 2021 by the August 2021 value.

**Table 1:** Coordinates used to extract shipping density data for comparison between 2021 and 2022. Data were extracted from a 20km radius around these points.

Port region	Longitude	Latitude
Odesa	30.74	46.50
Mariupol	38.14	46.97
Constanta	28.74	44.07

Mapping shipping route density was undertaken by downloading country boundaries from the ‘rnaturalearth’ package [43]. These were plotted around the shipping density data with the ‘ggplot2’ package with manual addition of country and location names using the ‘annotate’ function in ‘ggplot2’. Spatial data for route density from January through August 2021 and 2022 were generated by converting the raster stack object to a two-dimensional dataframe, grouping observations by year, and summing route density values with the ‘tidyverse’ package. The resulting dataframe was then passed to a ‘raster’ plot argument in ‘ggplot2’, and year was added as a ‘facet_wrap’ argument.

All data and code used to produce the shipping analysis and plots are available in online R vignettes (https://mickwelli.github.io/Ukraine-crops/articles/Ukraine_ship_analysis.html) [38].

## Results and discussion

### Validation with real crop production data

**Fig 1:**
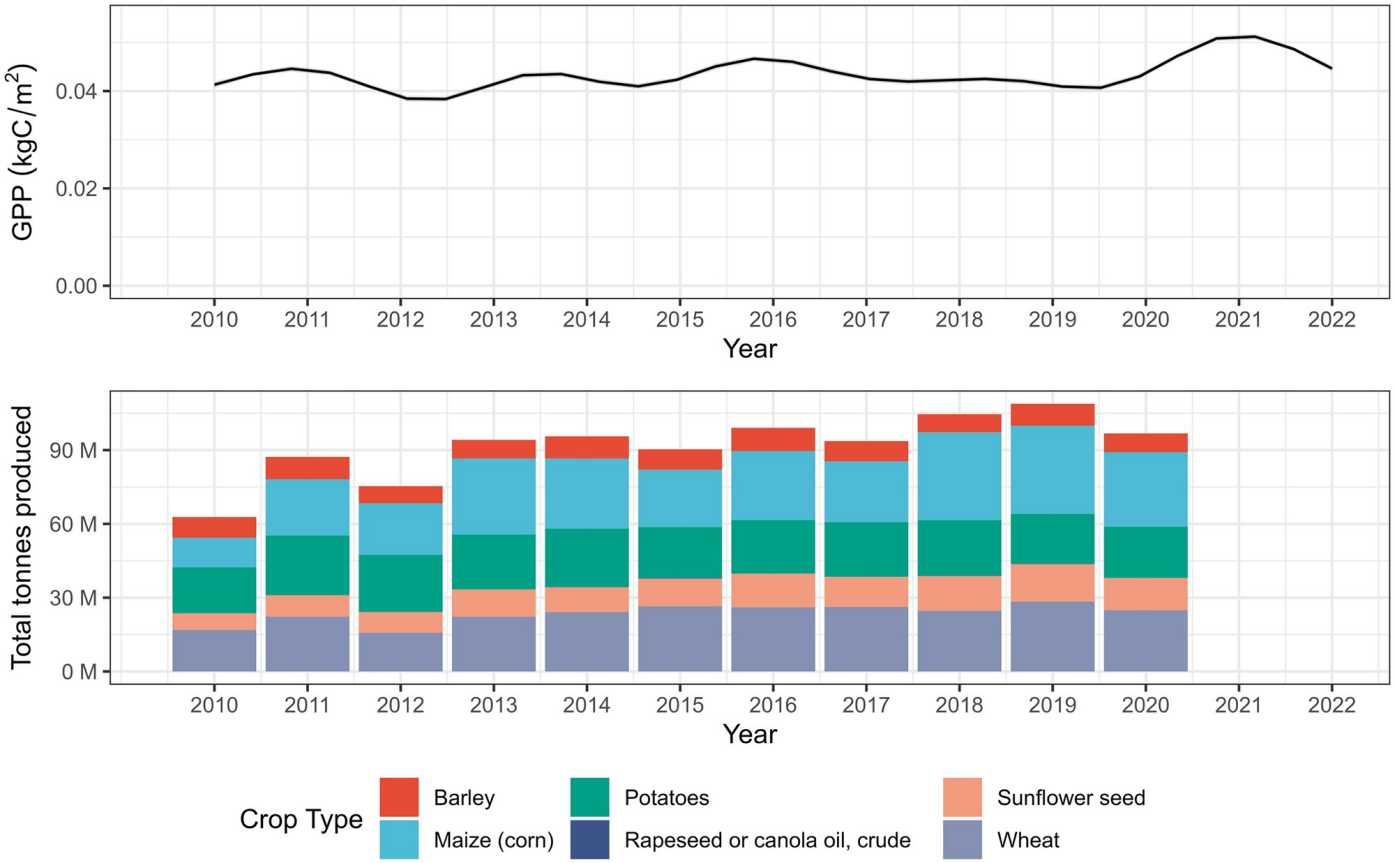
Summed effect of year on cropland Gross Primary Productivity (GPP) in Ukraine with a shaded ribbon, albeit visibly small, denoting the standard error (top). Values were extracted from a generalised additive model which decomposed the time series into seasonal (monthly) and annual trends and accounted for variation in space. This trend can be compared with annual total production volumes for major crops in Ukraine (bottom) taken from the FAOSTAT database [39].

The inter-annual trend in cropland GPP derived from a GAM, as a surrogate indicator of crop production, shows agreement with historical crop production in Ukraine (Fig 1). A one-sided Pearson correlation test for a positive relationship between total tonnes of major crop production and the model terms for year confirmed this agreement (r = 0.67, t = 2.74, df = 9, p = 0.012).

### Changes in cropland productivity post-invasion

Fitting ‘war’ as a factor in the GAM framework revealed cropland GPP to be significantly lower (-0.247gC/m^2^, t = -5.92, p = <0.001) following the invasion of Ukraine. This adds quantitative statistical inference to assessments of the Ukraine crop season from visual inspection of satellite image time series [16, 17].

**Fig 2:**
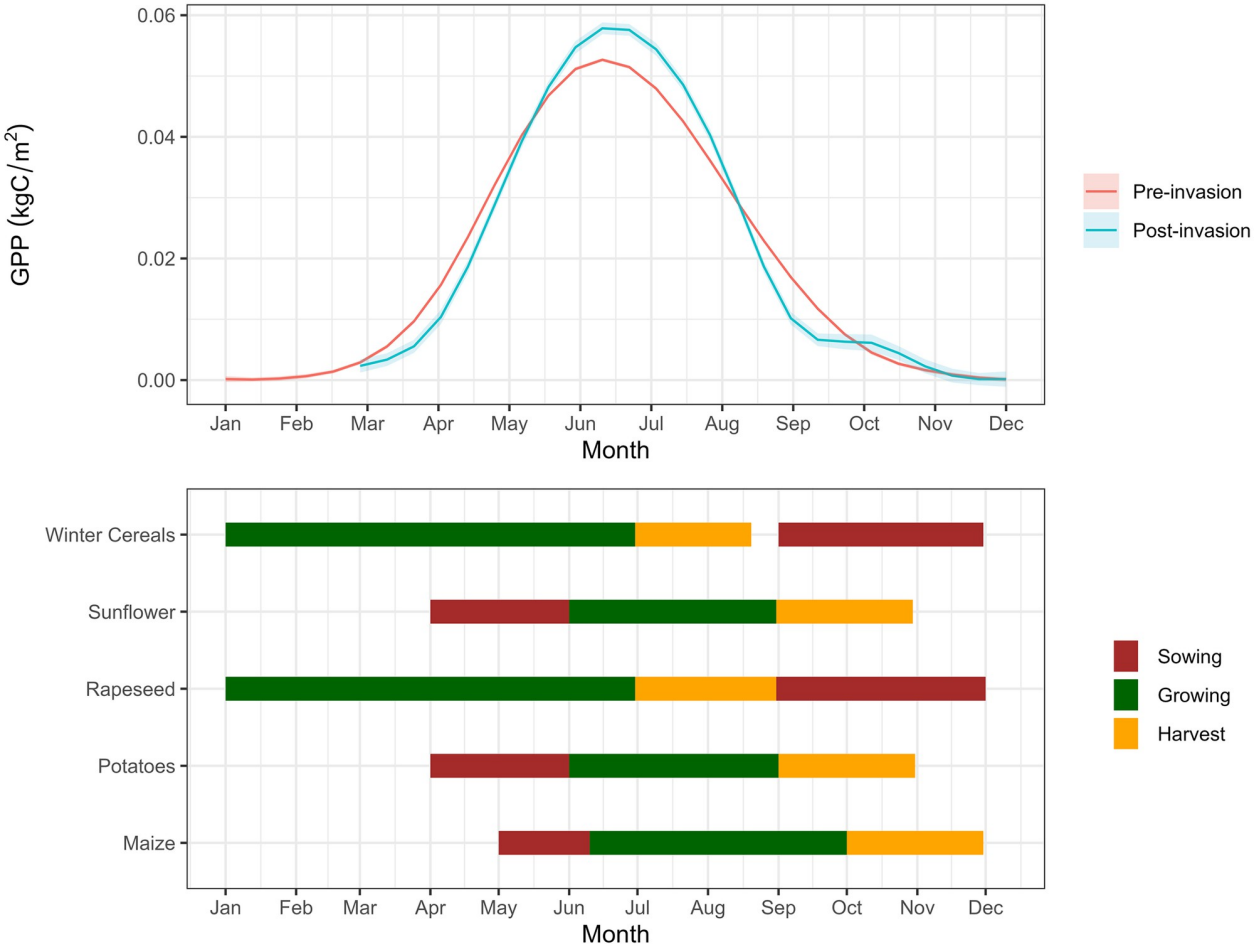
Summed effect of month on cropland Gross Primary Productivity (GPP) pre and post invasion of Ukraine with a shaded ribbon denoting the standard error. Estimates were derived from a GAM which decomposed the time series into seasonal (monthly) and annual trends, accounted for variation in space, and fitted invasion as a factor (top). Inferences can be drawn with reference to a crop calendar for Ukraine (bottom). The crop calendar was adapted from the UN Food and Agriculture Organisation Global Information and Early Warning System on Food and Agriculture [1].

The quantitative comparison of cropland GPP pre and post invasion can be complemented by visual assessment of crop season progression within the GAM statistical inference framework. Fig 2 shows that crops, especially winter cereals, may have been sown later than usual in the 2022 season. The later peak in GPP suggests maturity and harvest may have also been later than usual. The post-invasion GPP curve declined more rapidly than usual which may indicate a lower summer crop yield, increased cropland abandonment, or a change in the crop-type profile. Overall, these results align with media reports of continued crop management and harvest activity despite the conflict [6] and with other satellite and forecasting assessments that have predicted higher than average winter cereal yields prior to July 2022 [1, 16], but lower crop production beyond July 2022 [12].

**Fig 3:**
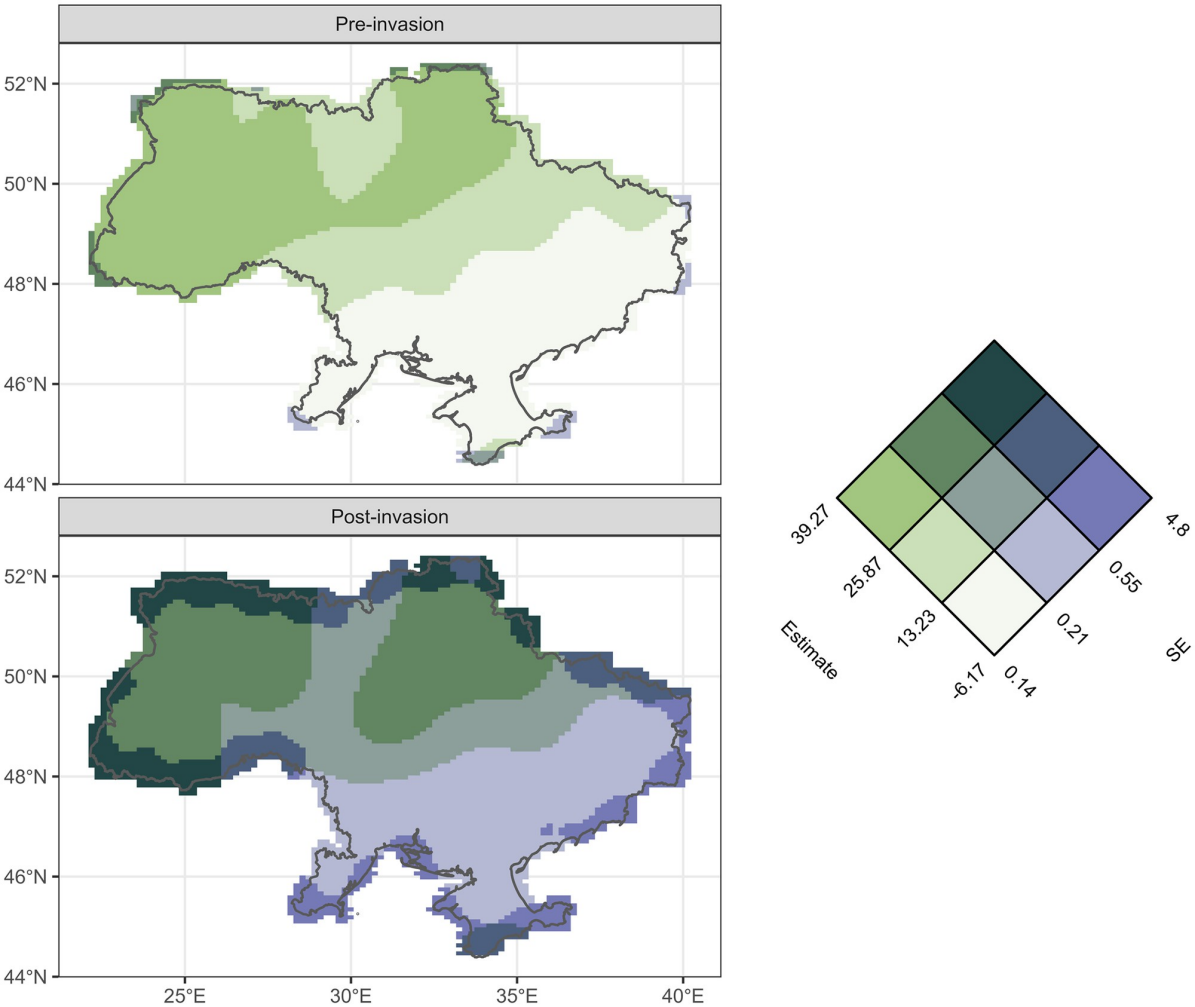
Predicted cropland Gross Primary Productivity (GPP) values for Ukraine pre and post invasion on the 24^th^ February 2022. A bivariate colour map is used to illustrate terciles of GPP estimates (green shades) in gC/m2 and standard errors (purple shades) associated with those estimates. Values were extracted from a generalised additive model which decomposed the time series into seasonal (monthly) and annual trends and accounted for variation in space. The Vizumap R package, which enables visualisation of uncertainty on maps, was used to generate the bivariate colour palette and maps [44]. Country bounds were taken from the rnaturalearth package [43].

Comparing the spatial distribution of cropland productivity pre and post invasion can identify specific regions of lower or higher crop productivity. Fig 3 shows GPP estimates (green) and their associated uncertainty (purple) for the entirety of Ukraine pre and post invasion. There is a similar overall distribution, with higher GPP values in the west of Ukraine for both periods. Post-invasion spatial estimates are associated with greater uncertainty due to the lower number of satellite images from the shorter time period contributing to the estimate.

### Changes in cargo shipping post-invasion

Complementary analyses of cropland GPP with assessment of other parts of the supply chain, such as export cargo shipping, provides a more holistic picture of value chain performance. The impact of Black Sea port closures on global food trade have been well described [3, 45]. Their reopening should mean that grain and other goods can resume flowing to importing nations [9]. However, ensuring the reopening deal is honoured and transporting large volumes of backlogged grain remains a challenge [46]. Rapid monitoring of cargo shipping activity enables institutions to quickly inspect spatiotemporal cargo route patterns and infer progress of grain exports.

**Fig 4:**
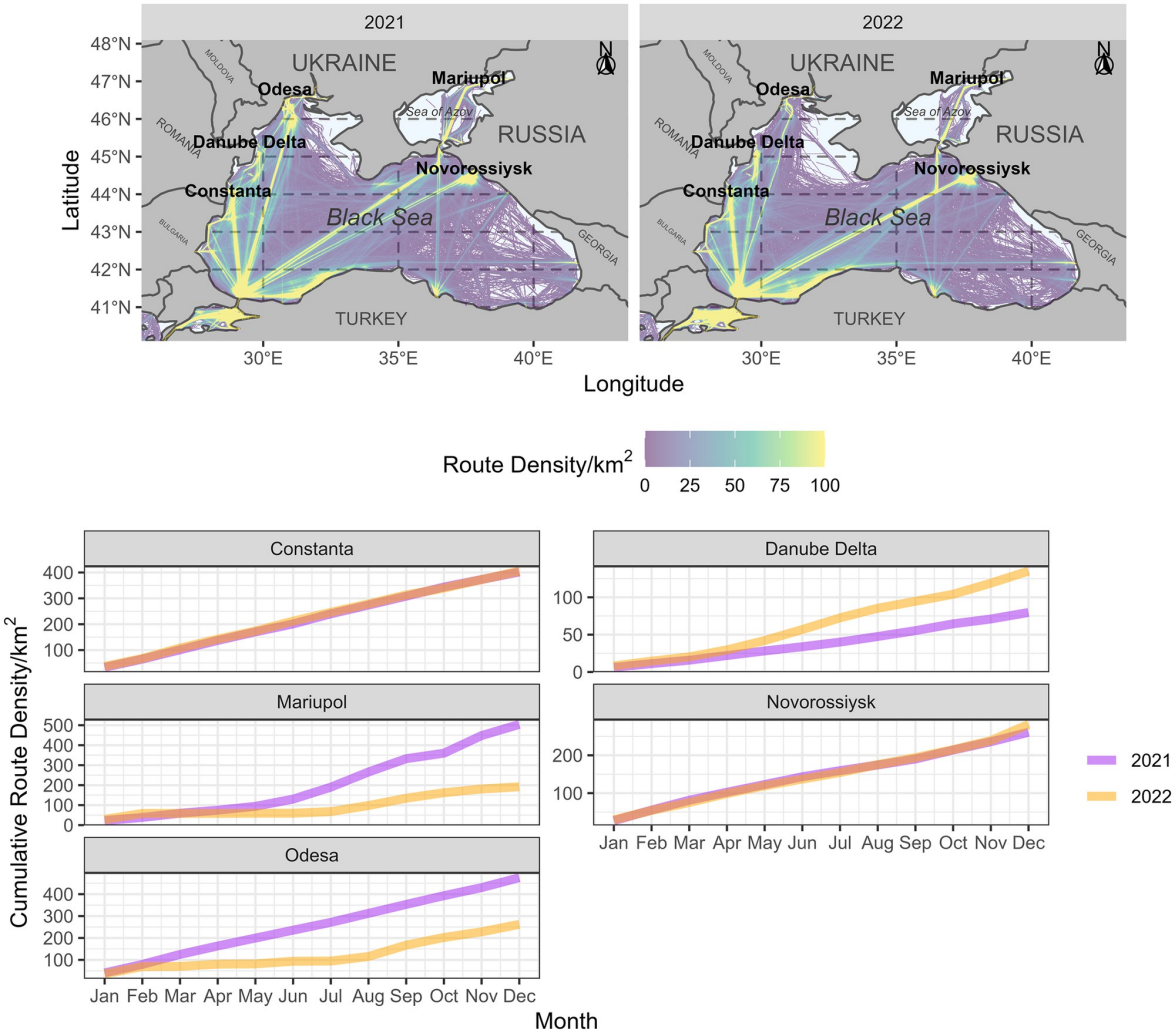
Cargo shipping route density through the Black Sea between 2021 and 2022 (top). Countries and major port centres are labelled, many of which have several individual ports, such as at Odesa. Higher density routes are highlighted by lighter shades towards yellow as depicted by the legend, and white in the ocean regions indicates absence of shipping. Cumulative route density for 2021 versus 2022 (bottom) was derived from extracting the mean route density from a 20km radius around each port zone. Data was collected from the European Marine Observation and Data Network [41]. Country bounds were taken from the rnaturalearth package [43].

Comparing cargo ship route densities (Fig 4, top) between January to August 2021 and 2022 shows that activity around Ukrainian ports at Odesa and Mariupol decreased in 2022 relative to 2021. Furthermore, comparing the cumulative route density shows that this can be attributed to effectively zero activity from February to June (Fig 4, bottom), with some activity evident from July. The cumulative route density to August 2022 at both the Odesa and Mariupol port zones were 45% and 62% in 2022 than in 2021, respectively. Ongoing monitoring of these data will reveal whether shipping activity recovers into 2023.

Cumulative cargo shipping route density in 2022 at the Danube River Delta was 70% greater than in 2021 (Fig 4), contrasting Mariupol and Odesa. This confirms media reports of Ukrainian grain being exported through previously disused ports along the Danube [47]. There have also been reports and evidence of Ukrainian grain entering the global market from the Constanta port in Romania [48]. While shipping to and from Constanta through the Black Sea has remained active throughout 2022, cumulative route density in 2022 appears very similar to 2021 (Fig 4). Fig 4 shows that Ukrainian ports represented a large volume of cargo traffic through the Black Sea in 2021 and it is therefore unlikely that alternative ports can adequately compensate for their lack of operation.

### Food security implications

The visualisation of shipping data also reflects changes in fertiliser exports from Ukraine, which, like grain, contribute greatly to fertiliser imports in some African nations such as Malawi [3, 5]. Disruptions to shipping in the Black Sea therefore impact food security in vulnerable nations through two compounding effects: reducing supply of grain imports and increasing food prices, and limiting the economic feasibility of filling the deficit through domestic grain production due to increased fertiliser prices [45].

More broadly, the greater vulnerability of export logistics to conflict compared with the relative resilience of the farm sector can inform future responses to food supply issues during conflict. Some studies on the impacts of conflict on food systems have focused on farm production [8, 49]. However, our analysis suggests that disruption to supply and logistics may be a greater threat. This could be due to reliance on logistics facilities in a few key locations [3], which contrasts the geographically diverse and large number of enterprises that comprise the farm sector. Following Bentley et al. [3] we suggest that investing in and securing supply chain resilience should be prioritised during conflict, without neglecting the farm sector. This could be achieved by garnering international support for the security of key ports during conflict, as the United Nations has endeavored to attain in Ukraine [9].

### Methodological implications

The application of low and moderate resolution analysis introduces some limitations to the detail of inference possible. Primary productivity is an incomplete and imperfect proxy for crop production, which is ultimately determined by sown area and yield. Crop-type specific sown area and yield forecasting analyses, such as those conducted for wheat in Ukraine by Kogan et al. [50], Deininger et al. [51], and Kussul et al. [52] are necessary to accurately estimate total production at local scales. Current initiatives to produce global scale crop-type maps, such as the ESA World Cereal project, are likely to enable more accurate remote sensing analyses for specific commodities, such as wheat. They may also enable differentiation of production changes into both contraction of cropped area and change in yield, drawing on annual active cropland maps.

However, we have demonstrated that surrogate indicators (Fig 1) allow rapid inference of inter-annual and seasonal patterns to allow decision-maker preparedness and lead to more detailed investigations and analysis. The application of novel computation statistical modelling that is capable of accommodating gigabytes of data [18, 19] and at scale, enables useful spatial and temporal inferences to be drawn from satellite image time series in an instant (Figs 1–4). Due to the global nature of remote sensing datasets available on platforms such as Google Earth Engine, this approach can be easily scaled to other regions experience disturbances due to climatic influences or conflict, such as Sudan in 2023.

Additionally, we have shown that monitoring shipping activity can identify adaptive measures, such as redirecting exports through the Danube. This method can also be used to monitor the progress of shipping into 2023 following the deal to reopen ports [9, 46]. Therefore, the approaches detailed in this analysis are particularly important for governments and international agencies who need to take immediate action and respond to food value chain disruptions quickly.

## Conclusions

Broad, satellite-based assessment of cropland productivity in Ukraine suggest that cropland GPP was significantly lower in 2022 compared with a historical baseline. Furthermore, explorative assessment of shipping routes demonstrates that there has been severe disruption of export logistics. Ensuring efficient passage of Ukrainian crop products to the global market will be critical to ensuring food security in vulnerable nations. The rapid monitoring approaches presented here demonstrate the potential to employ statistically robust inference techniques on very large datasets to monitor global challenges.
